# Large-Scale Profiling of Coding and Long Noncoding Transcriptomes in the Hippocampus of Mice Acutely Exposed to Vaporized CBD or THC

**DOI:** 10.3390/ijms26157106

**Published:** 2025-07-23

**Authors:** Mi Ran Choi, Jihun Kim, Chaeeun Park, Seok Hwan Chang, Han-Na Kim, Yeung Bae Jin, Sang-Rae Lee

**Affiliations:** 1 Laboratory Animal Research Center, Ajou University School of Medicine, Suwon 16499, Republic of Korea; mrchoi2007@ajou.ac.kr; 2 Efficacy Test Center for Mental & Behavioral Disorders, Ajou University Hospital, Suwon 16499, Republic of Korea; soulmate415@ajou.ac.kr (J.K.); smilechanni@ajou.ac.kr (C.P.); tjrghksekrzj@ajou.ac.kr (S.H.C.); 3 Department of Pharmacology, Ajou University School of Medicine, Suwon 16499, Republic of Korea; 4 College of Veterinary Medicine, Gyeongsang National University, Jinju 52828, Republic of Korea; hnkim@gnu.ac.kr

**Keywords:** cannabidiol, delta-9-tetrahydrocannabinol, hippocampus, transcriptome, vaping

## Abstract

Cannabis vaping, particularly involving cannabidiol (CBD) and delta-9-tetrahydrocannabinol (THC), rapidly delivers highly concentrated cannabinoids to the brain, potentially affecting the hippocampus. This study examined differential expression of long noncoding RNAs (lncRNAs) and mRNAs in the hippocampus after acute exposure to vaporized CBD or THC. Male ICR mice were exposed to vaporized CBD or THC (50 mg, n = 5/group), and hippocampal tissues were collected at 1, 3, and 14 days post-exposure. Total RNA sequencing was conducted on day 1 samples, and selected transcripts were validated using qRT-PCR across multiple time points. CBD led to significant up- or downregulation of *L3mbtl1*, *Wnt7a*, and *Camk2b* at day 1. However, *Wnt7a* showed gradual recovery at days 3 and 14. In the THC group, *Grin2a*, *Gria3*, and *Golga2* were significantly upregulated, while *Drd1*, *Drd2*, *Gnal*, and *Adcy5* were significantly downregulated at day 1. Time-course analysis showed that *Drd2* expression returned to baseline by day 14, whereas *Adcy5* remained persistently downregulated through days 3 and 14. In the CBD group, *NONMMUT069014.2* was upregulated, while *NONMMUT033147.2* and *NONMMUT072606.2* were downregulated at day 1; notably, *NONMMUT072606.2* showed a transient increase at day 3 before returning to baseline. In the THC group, *NONMMUT085523.1* and *NONMMUT123548.1* were upregulated, whereas *NONMMUT019734.2*, *NONMMUT057101.2*, and *NONMMUT004928.2* were downregulated, with most showing gradual recovery by day 14. Correlation analysis revealed that THC-responsive lncRNAs—including *NONMMUT004928.2*, *NONMMUT057101.2*, and *NONMMUT019734.2*—were strongly associated with downregulated mRNAs such as *Drd2* and *Adcy5*. These findings highlight cannabinoid-specific hippocampal transcriptomic responses and suggest potential regulatory roles for lncRNA–mRNA interactions in cannabinoid-induced neural changes.

## 1. Introduction

The rising popularity of cannabis products, particularly through vaping, has become a significant public health concern due to their links to various neurological and societal issues [1]. Unlike traditional cannabis consumption, vaping cannabis oil delivers high concentrations of active cannabinoids, such as delta-9-tetrahydrocannabinol (THC) and cannabidiol (CBD), rapidly and efficiently. This has led to increased recreational use, particularly among adolescents and young adults. Vaping cannabis has been associated with impaired cognitive function, memory deficits, and altered emotional regulation, as well as acute respiratory issues, including vaping-associated lung injury (e-cigarette- or vaping-product-use-associated lung injury, EVALI) [1,2,3]. The accessibility and perceived safety of vaping have contributed to its widespread use, yet its potential effects on brain health remain largely unknown.

Cannabis produces over 100 cannabinoids, including the most abundant chemicals, CBD and THC. While CBD is recognized for its therapeutic and non-psychoactive properties, THC influences mood, perception, and memory [4,5]. However, when exposed to high temperatures during vaping, CBD can degrade into THC and harmful byproducts like benzene and formaldehyde [6,7]. The increasing prevalence of CBD vaping has raised concerns about its potential neurological impact, including cognitive impairment and susceptibility to substance use disorders [2]. THC exposure has been associated with changes in synaptic plasticity, neuroinflammation, and disruptions in reward pathways, contributing to addiction risk and mood disorders [1,3,8]. These effects are largely mediated through the endocannabinoid system, which consists of CB1 and CB2 receptors. CB1 receptors, primarily found in the central nervous system, interact with THC to regulate neurotransmission, appetite, and pain perception, whereas CB2 receptors, mainly located in immune cells and peripheral tissues, interact with CBD to modulate immune responses and inflammation [9,10]. Given these interactions and potential risks, further research is essential to understand how CBD and THC exposure through vaping affects brain function at molecular and genetic levels.

The hippocampus, which is rich in CB1 receptors, is involved in memory formation, learning, emotional regulation, and stress responses. Emerging evidence suggests that CBD exerts significant influence on hippocampal function and structure through multiple mechanisms [11,12,13]. Previous studies have demonstrated that CBD enhances adult hippocampal neurogenesis, which can explain its anxiolytic and antidepressant properties [12,14,15]. Furthermore, CBD has been shown to increase cerebral blood flow specifically in the hippocampus, suggesting a direct modulatory effect on this region [8]. On the other hand, activation of CB1 receptors in the hippocampus by cannabinoids like THC can disrupt processes essential for memory storage, leading to impairments in short-term memory [16,17]. Acute exposure to THC has been shown to impair hippocampal function in healthy individuals, indicating that even a single dose of THC can transiently disrupt memory processes and alter hippocampal activity [18]. Understanding how cannabis affects the hippocampus is crucial for comprehending its impact on cognitive functions and the development of addictive behaviors.

Despite growing insights into the physiological and behavioral effects of cannabinoids, transcriptomic investigations have largely centered on peripheral organs such as the heart [19], liver [20], and kidney [21] or whole-organism developmental models like zebrafish [22,23]. These studies have revealed consistent modulation of genes involved in oxidative stress response, immune signaling, and metabolic regulation, many of which are also critically active in the hippocampus. Notably, Philippot et al. [24] demonstrated that a single developmental exposure to THC in mice altered hippocampal expression of neurotrophic and apoptotic markers, including *Trkb* and *Bax*, implicating early transcriptional changes as key events in cannabinoid-induced neurotoxicity. However, comprehensive transcriptomic profiling of the hippocampus in response to cannabinoids, especially using high-throughput RNA sequencing, remains limited. In particular, long noncoding RNAs (lncRNAs) are RNA transcripts longer than 200 nucleotides that do not encode proteins but play crucial regulatory roles in various cellular processes. They have emerged as important regulators of gene expression, chromatin remodeling, and RNA stability [25,26,27]. In the brain, lncRNAs are involved in neuronal differentiation, synaptic transmission, and responses to neuroinflammation [26,27,28]. Their region- and cell-type-specific expression patterns suggest that they help fine-tune gene regulatory networks in the hippocampus. Given the hippocampus’s enrichment in CB1 receptors and its essential role in neuroplasticity and cognitive function, transcriptome-wide approaches are necessary to elucidate the molecular basis of cannabinoid effects in this region. Furthermore, the inclusion of lncRNAs, which are known to play regulatory roles in neuronal development, synaptic plasticity, and neuroinflammation [27,29], might offer novel insights. Previous lncRNA–mRNA co-expression studies have identified key regulatory axes in contexts such as kidney aging [29], neurotoxicity [27], and cancer [30], yet their relevance in hippocampal cannabinoid response has not been explored. Therefore, applying total RNA sequencing (RNA-seq) to profile both mRNAs and lncRNAs in the hippocampus following cannabinoid exposure is a crucial step toward identifying novel biomarkers and decoding the transcriptional programs that underlie cannabinoid-related neurocognitive changes.

We hypothesized that a single exposure to CBD or THC via vaping induces distinct and time-dependent transcriptomic changes in the hippocampus, including both mRNA and lncRNA changes, which might underlie cannabinoid-induced neurobiological effects. Therefore, we analyzed the expression profiles of mRNAs and lncRNAs in the hippocampus of ICR mice following acute exposure to vaporized CBD or THC to explore their regulatory networks. Differentially expressed transcripts were functionally annotated, and selected targets were validated using quantitative RT-PCR (qRT-PCR). This study reveals novel cannabinoid-responsive transcripts and regulatory networks, providing insight into the molecular effects of cannabis vaping and aiding in the identification of biomarkers associated with hippocampal dysfunction.

## 2. Results

### 2.1. Histological Assessment of Hippocampal Changes Following Single Exposure to CBD or THC

**Figure 1:**
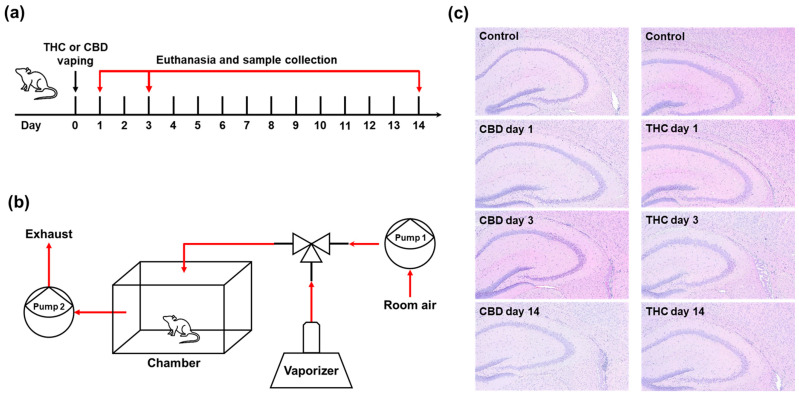
Histological assessment of hippocampal changes following single exposure to CBD or THC. (**a**) Experimental timeline of vapor exposure and tissue collection. (**b**) Custom-designed whole-body cannabis vapor exposure system. (**c**) Representative H&E-stained coronal sections of the hippocampus at days 1, 3, and 14 after a single exposure to vaporized CBD or THC. No significant morphological changes were observed in the hippocampus across control and treatment groups.

To investigate the time-dependent effects of a single exposure to vaporized cannabinoids on hippocampal gene expression, mice were subjected to acute inhalation of THC or CBD vapor. Following exposure, animals were sacrificed at designated time points (1, 3, or 14 days) (Figure 1a). A custom-designed whole-body exposure system was used to deliver vaporized cannabinoids in a controlled manner (Figure 1b). To assess histological alterations in the hippocampus following cannabinoid exposure, hematoxylin and eosin (H&E) staining was performed on brain sections collected at each time point (Figure 1c). Histological examination revealed no significant morphological changes, cellular damage, or overt signs of neurotoxicity in the hippocampus of mice exposed to either CBD or THC compared to control animals. Across all groups and time points, hippocampal cytoarchitecture, including the dentate gyrus and cornu ammonis regions, remained intact with no observable neuronal loss, gliosis, or structural disruption. Despite the absence of gross morphological changes, we posited that cannabinoid exposure could induce transcriptomic alterations.

### 2.2. Differential Expression Profiling of mRNAs and lncRNAs in the Hippocampus After CBD or THC Exposure

**Figure 2:**
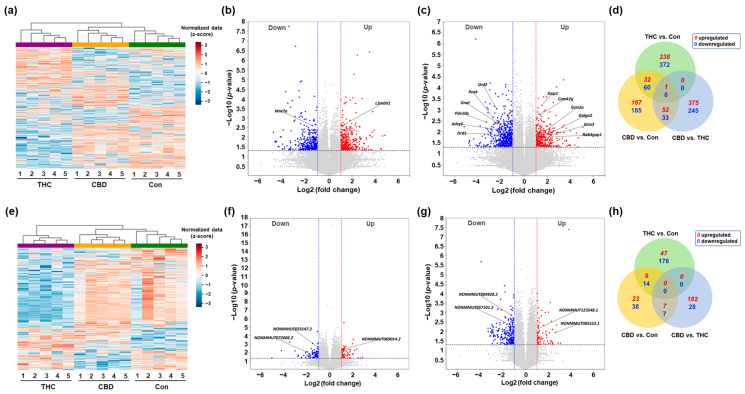
Differential expression of mRNAs and lncRNAs in the hippocampus at day 1 following single exposure to CBD or THC. (**a**) Heatmap showing hierarchical clustering of differentially expressed mRNAs among control (Con), CBD, and THC groups. (**b**,**c**) Volcano plots showing significantly upregulated (red) and downregulated (blue) mRNAs in the CBD (**b**) and THC (**c**) groups compared to the Con group. (**d**) Venn diagram depicting the overlap of differentially expressed mRNAs between CBD and THC groups. (**e**) Heatmap of differentially expressed lncRNAs among groups. (**f**,**g**) Volcano plots showing significant alterations in the CBD (**f**) and THC (**g**) groups relative to the Con group. (**h**) Venn diagram illustrating the overlap of differentially expressed lncRNAs between groups.

To investigate the transcriptomic alterations in the hippocampus following a single exposure to vaporized CBD or THC, we performed total RNA-seq and analyzed the transcript expression profiles using ExDEGA (Excel-based differentially expressed gene analysis; Ebiogen Inc., Seoul, Republic of Korea). Differentially expressed mRNAs and lncRNAs were then identified by applying thresholds for fold change (|log_2_ fold change| ≥ 1), expression level (log_2_ normalized expression ≥ 2), and statistical significance (*p*-value < 0.05), as described in the Methods. Subsequent multiple-testing correction using the Benjamini–Hochberg false discovery rate (FDR) method confirmed that all initially identified differentially expressed mRNAs and lncRNAs maintained statistical significance (adjusted *p*-value < 0.05). Hierarchical clustering analysis revealed distinct expression patterns among the CBD, THC, and control groups, illustrated by heatmaps (Figure 2a,e). mRNAs differentially expressed in CBD and THC groups, respectively, compared to the Con group, were visualized using volcano plots (Figure 2b,c). In the CBD group, representative upregulated genes included *L3mbtl1*, while *Wnt7a* was among the significantly downregulated genes (Figure 2b). Similarly, in the THC group, genes such as *Dzip1*, *Grin2a*, *Golga2*, and *Rab3gap1* were significantly upregulated, whereas *Drd1*, *Drd2*, and *Adcy5* were notably downregulated (Figure 2c). To further compare the overlap and uniqueness of differentially expressed mRNAs between treatment groups, a Venn diagram analysis was conducted (Figure 2d). The results demonstrated that most differentially expressed mRNAs were uniquely regulated in each condition, with a relatively small number of mRNAs commonly altered across both CBD and THC treatments. In parallel, differentially expressed lncRNAs were analyzed. Hierarchical clustering of differentially expressed lncRNAs showed distinct expression patterns between CBD, THC, and control groups (Figure 2e), with the CBD and control groups displaying similar expression profiles, in contrast to the markedly distinct pattern observed in the THC group. Volcano plots of differentially expressed lncRNAs revealed significant upregulation of *NONMMUT069014.2* in the CBD group and *NONMMUT123548.1* in the THC group, while *NONMMUT033147.2*, *NONMMUT072606.2*, *NONMMUT004928.2*, and *NONMMUT085523.1* were among the downregulated transcripts (Figure 2f,g). A Venn diagram comparing differentially expressed lncRNAs across groups showed minimal overlap, suggesting that CBD and THC modulate largely distinct sets of lncRNAs in the hippocampus (Figure 2h).

### 2.3. GO Enrichment and KEGG Pathway of Differentially Expressed mRNAs in the Hippocampus Following CBD or THC Exposure

**Figure 3:**
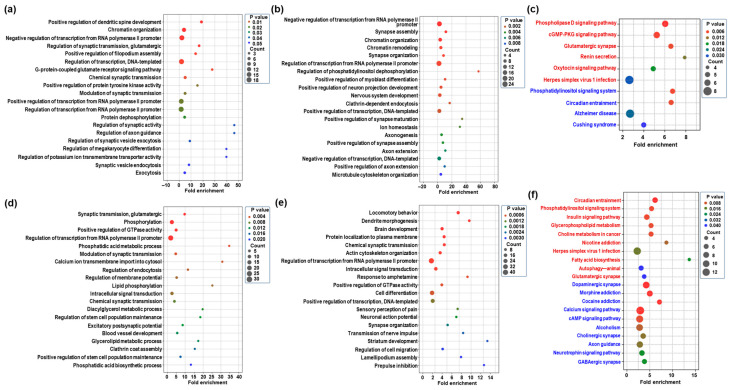
Classification of differentially expressed mRNAs in the CBD or THC group compared to the control group as assessed using gene ontology (GO) and Kyoto Encyclopedia of Genes and Genomics (KEGG) pathway enrichment. (**a**,**b**) The top 20 enriched GO terms in the biological process (BP) category for upregulated (**a**) and downregulated (**b**) mRNAs in the CBD group compared to the control group. (**c**) KEGG pathway enrichment analysis of upregulated (red) and downregulated (blue) mRNAs in the CBD group. The dot plot displays only those KEGG pathways that were significantly enriched (*p* < 0.05). (**d**,**e**) The top 20 enriched GO terms in the BP category for upregulated (**d**) and downregulated (**e**) mRNAs in the THC group compared to the control group. (**f**) KEGG pathway enrichment analysis of upregulated (red) and downregulated (blue) mRNAs in the THC group.

To elucidate the biological functions associated with transcriptomic changes in the hippocampus following CBD or THC exposure, we analyzed the differentially expressed mRNAs using gene ontology (GO) and Kyoto Encyclopedia of Genes and Genomes (KEGG) pathway enrichment analyses. The GO analysis primarily focused on the biological process (BP) category. Table S1 presents significantly enriched BP terms and KEGG pathways (*p* < 0.05) for each comparison group. In the CBD group, upregulated mRNAs were significantly enriched in BP terms related to chromatin organization and chemical synaptic transmission (Figure 3a and Table S1). In contrast, downregulated mRNAs were associated with synapse assembly and nervous system development (Figure 3b and Table S1). KEGG pathway analysis further revealed that CBD exposure led to the phospholipase D signaling pathway and a cGMP-PKG signaling pathway, while downregulated pathways included a phosphatidylinositol signaling system and circadian entrainment (Figure 3c and Table S1). In the THC group, upregulated mRNAs were enriched in BP terms involved in synaptic transmission, glutamatergic excitatory postsynaptic potential, and neuromuscular synaptic transmission (Figure 3d and Table S1). Downregulated genes were significantly associated with processes including dendrite morphogenesis, brain development, and response to amphetamine (Figure 3e and Table S1). KEGG pathway analysis showed that upregulated pathways included circadian entrainment, insulin signaling, and glutamatergic synapses, while downregulated pathways involved dopaminergic, GABAergic, and cholinergic synapses, as well as multiple addiction-related pathways such as morphine and cocaine addiction (Figure 3f and Table S1). These results suggest that CBD and THC induce distinct transcriptomic responses in the hippocampus, with THC exerting a broader influence on synaptic and addiction-related pathways.

### 2.4. Prediction and Visualization of mRNA-lncRNA Interaction Networks in Response to CBD and THC Exposure

**Figure 4:**
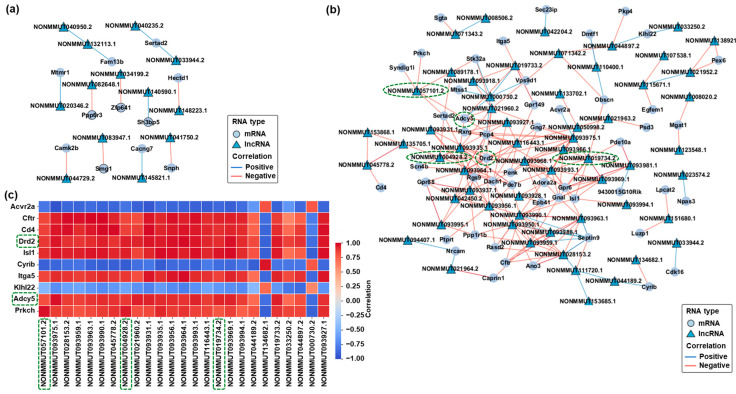
Co-expression networks and correlation heatmap of differentially expressed mRNAs and lncRNAs in the hippocampus following CBD or THC exposure. (**a**) Co-expression network of differentially expressed mRNAs and lncRNAs in the CBD group compared to the control group. (**b**) Co-expression network of differentially expressed mRNAs and lncRNAs in the THC group compared to the control group. mRNA–lncRNA pairs outlined with green dashed boxes represent concordantly downregulated transcripts with strong positive correlations, which were selected for further qRT-PCR validation. (**c**) Correlation heatmap of selected mRNA–lncRNA pairs in the THC group. The color scale represents the correlation strength ranging from −1 (perfect negative correlation, blue) to 1 (perfect positive correlation, red). mRNA–lncRNA pairs outlined with green dashed boxes indicate concordantly downregulated transcripts with strong positive correlations, corresponding to those selected for qRT-PCR validation.

To further explore the potential regulatory relationships between differentially expressed mRNAs and lncRNAs, interaction networks were constructed based on predicted binding affinities and co-expression patterns. In the CBD group, a set of pairs of differentially expressed mRNAs and lncRNAs meeting the filtering criteria formed a co-expression network (Figure 4a). These interactions were selected based on sequence complementarity and strong positive correlation coefficients. In the THC group, a more complex and extensive mRNA–lncRNA interaction network was observed (Figure 4b). Several differentially expressed lncRNAs, including *NONMMUT004928.2*, *NONMMUT057101.2*, and *NONMMUT019734.2*, showed strong positive correlations with downregulated mRNAs such as *Drd2*, *Adcy5*, and *Gnal*. To evaluate the consistency of these relationships, a correlation heatmap was generated based on expression profiles (Figure 4c). Most mRNA–lncRNA pairs exhibited strong positive correlations (Spearman ρ ≥ 0.95), supporting the robustness of the predicted interactions. Importantly, there were several concordantly downregulated pairs with high correlation values, as highlighted with green dashed boxes in the network and heatmap (Figure 4b,c). These pairs were selected for further validation via qRT-PCR and are considered promising candidates for future functional analysis and biomarker discovery in cannabinoid-induced hippocampal transcriptomic alterations. Our analysis primarily focused on positively correlated mRNA–lncRNA pairs based on their strong correlation coefficients and predicted binding affinities.

### 2.5. Validation of Selected mRNAs Based on qRT-PCR

**Figure 5:**
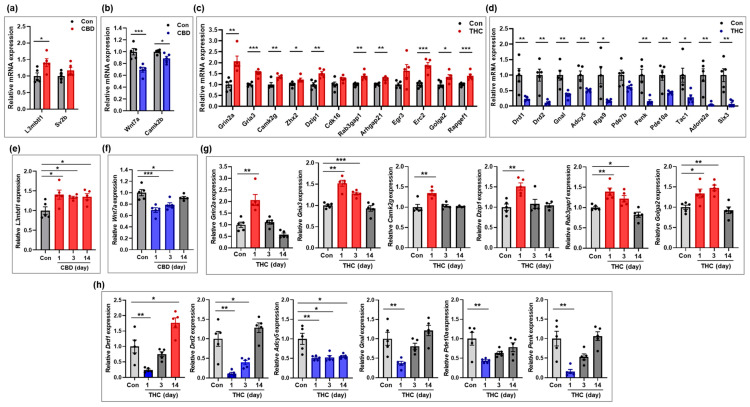
Validation of mRNAs changed in the hippocampus following CBD or THC exposure. (**a**,**b**) mRNAs that were upregulated (**a**) and downregulated (**b**) in the CBD group (n = 5) at day 1 compared to the control group (n = 5). (**c**,**d**) mRNAs that were upregulated (**c**) and downregulated (**d**) in the THC group (n = 5) at day 1 compared to the control group (n = 5). (**e**,**f**) Temporal expression patterns of mRNAs that were upregulated (**a**) or downregulated (**b**) at day 1 in the CBD group. Gene expression was examined at days 3 and 14 post-exposure to assess time-dependent changes (n = 5 per group). (**g**,**h**) Temporal expression patterns of mRNAs that were upregulated (**c**) or downregulated (**d**) at day 1 in the THC group. Gene expression was examined at days 3 and 14 post-exposure to assess time-dependent changes (n = 5 per group). * *p* < 0.05, ** *p* < 0.01, and *** *p* < 0.001.

To validate the expression of mRNAs identified in the transcriptomic analysis, four genes from the CBD group and 23 genes from the THC group were selected for qRT-PCR. Expression levels of some of these genes were further examined in hippocampal tissue at days 3 and 14 post-exposure, in addition to day 1. In the CBD group, *L3mbtl1*, a gene associated with chromatin organization, was significantly upregulated compared to the control group, and this upregulation persisted through days 3 and 14 (Figure 5a,e). In contrast, *Wnt7a*, involved in synapse assembly, and *Camk2b*, involved in nervous system development, were significantly downregulated at day 1 (Figure 5b). However, *Wnt7a* showed a gradual recovery at days 3 and 14, approaching expression levels comparable to those of the control group (Figure 5f). In the THC group, the genes *Grin2a*, *Gria3*, and *Camk2g* (associated with glutamatergic signaling); *Zhx2* and *Dzip1* (related to cell differentiation); *Rab3gap1* (involved in excitatory postsynaptic potential); and *Golga2* (related to axonogenesis) were significantly upregulated compared to the control group (Figure 5c and Table S1). Further analysis at days 3 and 14 post-exposure revealed that, with the exception of *Grin2a*—which was downregulated at day 14 compared to controls—most of these genes gradually returned to baseline expression levels over time (Figure 5g). Conversely, genes associated with dopaminergic synapses (*Drd1*, *Drd2*, *Gnal*, and *Adcy5*), sensory perception of pain (*Penk* and *Tac1*), brain development (*Six3*), and the cAMP signaling pathway (*Pde10a*) were significantly downregulated in the THC group compared to the control group (Figure 5d). Time-course analysis of some genes at days 3 and 14 showed that the expression of most of these genes recovered to control levels (Figure 5h), with the exception of *Drd1* and *Adcy5*. Notably, *Drd1* was upregulated at day 14, exceeding control levels, while *Adcy5* remained consistently downregulated across all time points. Overall, the qRT-PCR validation results were consistent with the transcriptomic profiles obtained from total RNA-seq at day 1, confirming the reliability of the sequencing data. Furthermore, the additional analyses at days 3 and 14 post-exposure provided insight into time-dependent changes in gene expression following a single exposure to THC or CBD.

### 2.6. Validation of Selected lncRNAs Based on qRT-PCR

**Figure 6:**
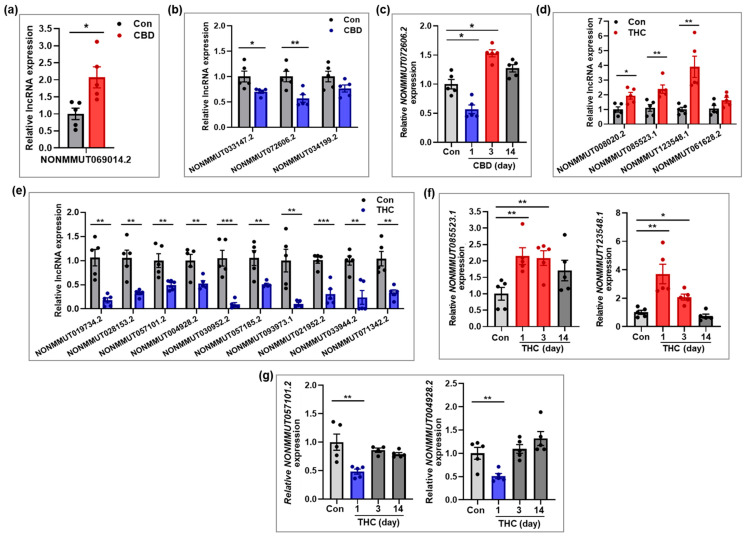
Validation of lncRNAs changed in the hippocampus following CBD or THC exposure. (**a**,**b**) lncRNAs that were upregulated (**a**) and downregulated (**b**) in the CBD group (n = 5) at day 1 compared to the control group (n = 5). (**c**) Temporal expression patterns of lncRNA that was downregulated in the CBD group at day 1 compared to the control group (n = 5). The lncRNA expression was investigated at days 3 and 14 post-exposure to assess time-dependent changes (n = 5 per group). (**d**,**e**) lncRNAs that were upregulated (**d**) and downregulated (**e**) in the THC group (n = 5) at day 1 compared to the control group (n = 5). (**f**,**g**) Temporal expression patterns of lncRNAs that were upregulated (**d**) or downregulated (**e**) at day 1 in the THC group. The lncRNA expression was examined at days 3 and 14 post-exposure to assess time-dependent changes (n = 5 per group). * *p* < 0.05, ** *p* < 0.01, and *** *p* < 0.001.

To validate the expression patterns of differentially expressed lncRNAs identified by RNA-seq, several lncRNAs from the CBD and THC groups were selected for qRT-PCR analysis. In addition to the analysis on day 1, expression levels of selected lncRNAs were further examined in hippocampal tissues at days 3 and 14 post-exposure to assess temporal expression changes. In the CBD group, *NONMMUT069014.2* was significantly upregulated at day 1 compared to the control group (Figure 6a), while *NONMMUT033147.2* and *NONMMUT072606.2* were significantly downregulated (Figure 6b). Interestingly, *NONMMUT072606.2*, which was downregulated at day 1, showed a significant increase in expression at day 3, before returning to baseline by day 14 (Figure 6c), suggesting a transient overcompensation effect. In the THC group, *NONMMUT085523.1* and *NONMMUT123548.1* were significantly upregulated, while *NONMMUT019734.2*, *NONMMUT057101.2*, and *NONMMUT004928.2* were significantly downregulated at day 1 (Figure 6d,e). Additional analysis of *NONMMUT123548.1* at days 3 and 14 revealed that its expression level, which was significantly upregulated at day 1, declined over time and either approached or fell slightly below baseline level by day 14 (Figure 6f). Similarly, the expression levels of *NONMMUT057101.2* and *NONMMUT004928.2*, which were significantly downregulated at day 1, recovered to control levels at day 3 (Figure 6g). Overall, the qRT-PCR validation results for lncRNAs were consistent with the RNA-seq findings at day 1 and further revealed dynamic, time-dependent expression changes following a single exposure to either CBD or THC.

## 3. Discussion

Cannabis vaping has gained popularity due to its efficient delivery of concentrated cannabinoids, but this mode of consumption has raised significant concerns regarding cognitive impairment, neuroinflammation, and hippocampal dysfunction [1,2,3]. THC and CBD, the primary cannabinoids of cannabis, interact with CB1 and CB2 receptors to modulate synaptic transmission and immune responses [9,10], and studies suggest that THC can impair memory processes while CBD enhances hippocampal neurogenesis and cerebral blood flow [8,12,14]. Despite these findings, transcriptomic profiling of the hippocampus following cannabinoid exposure remains scarce, especially with regard to lncRNAs, which are key regulators of neuronal development, synaptic plasticity, and neuroinflammation [26,27,29]. Given the high density of CB1 receptors and the critical role of the hippocampus in cognitive processes, we investigated large-scale mRNA and lncRNA expression changes following acute exposure to vaporized THC or CBD to elucidate the molecular basis of cannabis-induced hippocampal dysfunction.

In the present study, the GO and KEGG pathway analyses demonstrated that THC and CBD exert distinct molecular influences on hippocampal function. CBD exposure was associated with enrichment in pathways related to chromatin remodeling, signaling cascades, and circadian rhythm regulation, suggesting a more regulatory and modulatory transcriptomic profile. In contrast, THC exposure resulted in broader enrichment of pathways involved in synaptic activity, neuronal excitability, and multiple addiction-related signaling systems, including dopaminergic, glutamatergic, and GABAergic synapses. Notably, KEGG analysis revealed that several addiction-relevant pathways—such as those related to morphine, nicotine, and cocaine—were changed following THC exposure, implying a disruption of homeostatic neurotransmission potentially linked to vulnerability to substance use disorders [1,31]. These findings align with previous reports that THC interferes with neural signaling and reward circuits through its interaction with CB1 receptors [3,16], whereas CBD may exert stabilizing effects on transcriptional programs involved in brain homeostasis [8,11]. The functional enrichment patterns observed in this study underscore the divergent impacts of these two cannabinoids on hippocampal gene regulation. THC appears to induce widespread dysregulation of neuronal pathways, while CBD influences gene expression in a more targeted manner, potentially contributing to its reported neuroprotective properties. Taken together, these results suggest that even a single exposure to THC or CBD induces transcriptomic changes in hippocampal pathways that might influence cognitive and emotional processes; however, direct functional consequences remain to be established.

In our study, analysis of lncRNA–mRNA interactions revealed distinct regulatory networks underlying the transcriptomic divergence between CBD and THC exposure. Based on predicted binding affinities and co-expression patterns, we identified highly correlated lncRNA–mRNA pairs that suggest coordinated regulation. CBD exposure produced limited interaction patterns, reflecting selective modulation of hippocampal transcripts. In contrast, THC exposure elicited an intricate and densely connected network structure, with several lncRNAs acting as central hubs linked to genes involved in neurotransmitter signaling and synaptic plasticity. Of particular interest, key components of dopaminergic synapse and sensory perception of pain, such as *Drd2*, *Adcy5*, and *Penk*, were among the shared targets of correlated lncRNAs, implicating these transcripts in the regulation of neural signaling. These findings align with evidence suggesting that lncRNAs contribute to the fine-tuning of neural circuit dynamics in neuropsychiatric conditions [27,32]. Our analysis emphasized positively correlated mRNA–lncRNA pairs due to their robust correlation and predicted binding affinities; however, negatively correlated pairs indicating potential inverse regulatory relationships were not explicitly explored in this study. Future studies should examine both the positively and negatively correlated pairs to fully elucidate the complexity of these regulatory interactions and functionally validate their potential roles in cannabinoid-induced cognitive dysregulation.

Interestingly, our findings highlight the persistent upregulation of *L3mbtl1* expression in the hippocampus following a single exposure to vaporized CBD. The increased expression observed at 1 day post-exposure was sustained at the 3- and 14-day time points. L3mbtl1, lethal 3 malignant brain tumor-like protein 1, is involved in chromatin remodeling and transcriptional repression, functioning primarily as a transcriptional regulator through its histone-binding properties [33]. It has been implicated in epigenetic silencing mechanisms, particularly through its role in polycomb repressive complex 1 (PRC1), which influences gene expression critical to neural plasticity and memory formation [34,35]. The observed sustained elevation of *L3mbtl1* following CBD exposure suggests prolonged transcriptional regulatory effects, potentially stabilizing or repressing gene expression patterns linked to neural function and cognitive processes. However, further studies are required to determine whether this response represents a protective or adaptive mechanism. In addition, our results indicated significant downregulation of *Wnt7a* expression at 1 day post-CBD exposure, which recovered gradually at subsequent time points (3 and 14 days). Wnt7a is an essential modulator of synapse assembly and neural circuit connectivity, playing a critical role in synaptogenesis and synaptic plasticity [36]. The initial reduction followed by recovery in *Wnt7a* expression suggests a transient modulation of synapse-related processes following CBD exposure. The functional implications of this expression pattern require further clarification. This dynamic pattern aligns with previous reports on cannabinoid-induced modulation of Wnt signaling pathways, highlighting their role in neurodevelopmental processes and plasticity [37,38]. Thus, the transient modulation of *Wnt7a* by CBD exposure could represent a short-term adaptive response in synaptic function, contributing to the regulation of hippocampal homeostasis.

In the present study, a single exposure to THC resulted in a significant upregulation of *Grin2a* and *Gria3* at day 1, both of which are key components of glutamatergic synaptic transmission. *Grin2a* encodes the GluN2A subunit of the NMDA receptor, critical for synaptic plasticity and memory formation, while *Gria3* encodes the GluA3 subunit of the AMPA receptor, involved in rapid excitatory neurotransmission [39,40,41,42]. However, their expression levels gradually returned to baseline or showed a mild downward trend at 3 and 14 days post-exposure, suggesting a transient elevation rather than a sustained upregulation following THC exposure. This pattern may reflect an acute glutamatergic excitatory response to cannabinoid-induced disruption of hippocampal circuitry, followed by compensatory normalization. Previous studies have reported that cannabinoids, including THC, can transiently enhance glutamate release or receptor activity, particularly in the early phases of exposure, before inducing long-term depression-like effects on synaptic efficacy [41,42]. The transient upregulation of *Grin2a* and *Gria3* observed here might reflect an acute glutamatergic response following THC exposure. Further studies are needed to clarify the functional significance of these transient changes.

In this study, the cell differentiation-related genes *Camk2g*, *Zhx2*, and *Dzip1* were upregulated at day 1 following 50 mg THC exposure. *Camk2g* and *Dzip1* showed transient upregulation, suggesting an acute but reversible response. *Camk2g* regulates synaptic development via calcium signaling, while *Dzip1* is involved in Hedgehog signaling and ciliogenesis, which are critical for neurodevelopment [43,44]. These early changes suggest a transient response rather than a persistent alteration. Further research is necessary to determine the functional implications. Notably, since the current findings are based on a single 50 mg dose of THC, different concentrations or repeated exposures might induce more sustained or divergent gene expression patterns. Interestingly, *Golga2* expression was transiently elevated following THC exposure, showing significant upregulation on day 1 and remaining increased on day 3 before returning to baseline by day 14. *Golga2* encodes GM130, a cis-Golgi matrix protein essential for Golgi structure, vesicle tethering, and intracellular trafficking. In the nervous system, GM130 contributes to dendritic Golgi positioning, neuronal polarity, and axonogenesis, all of which are crucial for proper neurodevelopment [45,46,47]. The early increase in *Golga2* may reflect transient alterations affecting secretory and cytoskeletal pathways within neurons following THC exposure. However, the exact role of this response remains to be determined.

In line with previous findings that THC modulates dopaminergic and cAMP signaling pathways [1,42], our study identified significant transcriptomic changes in key hippocampal genes following acute exposure. Genes involved in dopaminergic signaling, particularly *Drd1*, *Drd2*, *Gnal*, and *Adcy5*—classified under the dopaminergic synapse pathway in KEGG pathway analysis—showed significant downregulation at day 1. These alterations likely reflect an immediate response to CB1 receptor activation, which has been shown to influence dopamine receptor expression and downstream signaling [48]. Time-course validation of these genes revealed distinct expression trajectories. *Drd1* expression showed a robust rebound by day 14, exceeding control levels, suggesting delayed neuroadaptive upregulation. In contrast, after initial downregulation, *Drd2* and *Gnal* gradually returned to baseline levels over time, indicating transient suppression followed by transcriptional recovery. *Adcy5* expression, however, remained persistently suppressed through days 3 and 14, suggesting long-term disruption of cAMP-mediated intracellular signaling. This observation is consistent with previous findings demonstrating that chronic THC exposure alters adenylate cyclase activity and reduces cAMP levels in limbic regions [49]. These gene-specific dynamics highlight the complex and heterogeneous molecular responses triggered by acute THC exposure, where some components of the dopaminergic system remain resilient, whereas others, such as *Adcy5*, exhibit prolonged vulnerability, underscoring the selective impact of cannabinoids on hippocampal signaling networks. Taken together, these findings reveal how acute THC exposure alters the expression of dopaminergic and intracellular signaling genes with both transient and lasting effects. Further studies are warranted to assess how repeated exposures might amplify or alter these molecular trajectories.

In this study, qRT-PCR validation of selected lncRNAs underscores their dynamic and regulatory potential following acute cannabinoid exposure. In the CBD group, the significant and immediate upregulation of *NONMMUT069014.2* at day 1 suggests an early transcriptional response potentially linked to protective or homeostatic mechanisms triggered by CBD. Conversely, the initial suppression of *NONMMUT033147.2* and *NONMMUT072606.2*, followed by a marked transient overcompensation in *NONMMUT072606.2* expression at day 3, indicates complex temporal regulatory dynamics. Such transient recovery patterns might represent compensatory molecular events aimed at restoring hippocampal homeostasis disrupted by initial cannabinoid perturbation, supporting the hypothesis that CBD induces nuanced, regulatory transcriptomic changes rather than prolonged disruptions. In contrast, THC exposure elicited distinctively broader and more temporally intricate lncRNA responses. The immediate upregulation of *NONMMUT085523.1* and *NONMMUT123548.1*, followed by normalization at subsequent time points, mirrors the acute yet transient pattern of associated mRNAs involved in neuronal differentiation and synaptic signaling pathways. Additionally, the significant initial downregulation of *NONMMUT057101.2* and *NONMMUT004928.2*, which recovered toward baseline levels at day 3, likely reflects regulatory adjustments aimed at mitigating sustained neural disruption induced by THC. Collectively, these findings substantiate the importance of lncRNAs as integral modulators of hippocampal gene networks in response to cannabinoid stimuli. Future studies should focus on functional characterization of these dynamically regulated lncRNAs to elucidate their precise roles in cannabinoid-induced neurobiological outcomes, providing valuable insights into their potential as therapeutic targets or biomarkers.

In summary, this study demonstrated distinct transcriptomic alterations in the hippocampus following a single exposure to vaporized CBD or THC, with THC eliciting broader and more extensive gene expression changes compared to CBD. The differential modulation of specific mRNAs and dynamically regulated lncRNAs indicates potential molecular pathways underlying cannabinoid-induced synaptic disruption, neuronal differentiation, and cognitive impairment. Correlation analysis further revealed significant regulatory relationships between lncRNAs and critical mRNAs, highlighting potential biomarkers and therapeutic targets relevant to cannabinoid-related neuropsychiatric dysfunction. However, our findings should be interpreted within the context of several limitations. First, transcriptomic profiling was conducted primarily at a single, early time point (one day post-exposure), limiting our understanding of the full temporal dynamics of gene expression changes. Second, this study utilized only male ICR mice exposed to a single dosage (50 mg) of cannabinoids. Future studies incorporating varied doses, repeated exposures, and female subjects could provide a more comprehensive understanding of cannabinoid impacts on hippocampal gene regulation. Third, a single control group was used for comparisons across multiple time points (days 1, 3, and 14 post-exposure), which assumes baseline transcriptomic levels remain stable throughout the two-week period. Although potential variability was minimized by controlling experimental conditions such as consistent sampling time-of-day and environmental factors, future studies should incorporate separate control groups at each sampling time point to more precisely account for baseline fluctuations in hippocampal gene expression. Lastly, functional characterization of identified lncRNA–mRNA interactions was not performed, and further investigations using knockdown or overexpression models are warranted to clarify their precise roles in cannabinoid-induced hippocampal dysfunction. Despite these limitations, this study provides novel molecular insights into the hippocampal response to cannabis vaping, laying important groundwork for future research on cannabinoid-induced neurobiological effects.

## 4. Materials and Methods

### 4.1. Animals

Thirty-five eight-week-old male ICR mice were obtained from KOATECH (Pyeongtaek, Gyeonggi-do, Republic of Korea) and were acclimated for one week prior to experimentation. Mice were individually housed in transparent plastic cages within individually ventilated cage systems under controlled conditions: 22 ± 2 °C temperature, 55 ± 10% relative humidity, and a 12 h light/dark cycle. Animals had free access to food and water throughout the study. Following acclimation, mice were randomly assigned to seven experimental groups (n = 5 per group, total n = 35).

### 4.2. Vaping Exposure

Each group was exposed to vaporized solutions in a custom-designed vaping chamber. The base vehicle consisted of a 7:1 mixture of propylene glycol (PG) and vegetable glycerin (VG). For the CBD group, 50 mg of CBD (Lipomed AG, Arlesheim, Switzerland) was dissolved in 4 mL of the PG/VG vehicle. THC (Lipomed) was dissolved in ethanol and then mixed into the same PG/VG base (7:1 ratio). The control group received PG/VG mixed with ethanol only, without cannabinoids. During exposure, the solution was vaporized for two seconds, followed by a 10 s inhalation period. This cycle was repeated 20 times (total duration: four minutes), followed by a six-minute forced ventilation period to clear residual vapor. Exposure was repeated until the vaping solution was depleted. Mice were sacrificed at 1, 3, or 14 days post-exposure. Control mice were sacrificed at 1 day post-exposure only and served as the baseline reference group for both histological and transcriptomic comparisons. A total of 50 mg of CBD or THC was vaporized as a single dose. To generate an animal model subjected to a single exposure of 50 mg CBD or THC through vaping, the dosage and exposure style were determined based on a previous study that developed an inhalation model in mice to mimic human cannabis vaping patterns [50].

### 4.3. Histological Analysis

Mice were euthanized with isoflurane, and samples were collected immediately. To minimize potential time-of-day bias, all animals were euthanized and sampled at 2:00 PM. On days when both control and treatment groups were processed, dissections were performed alternately (one control and one treated animal). Brain tissues were carefully extracted. Five 2 mm thick coronal sections from each brain were prepared with a commercial rat brain matrix (Acrylic brain matrix, Ted Pella, Inc., Redding, CA, USA). The tissues were fixed in 10% neutral buffered formalin, and the samples were processed through standard histological procedures: washing, graded ethanol dehydration, xylene clearing, and paraffin embedding. Using a rotary microtome, 3–5 µm thick paraffin sections were obtained. These sections were deparaffinized in xylene, rehydrated through graded ethanol, and stained with H&E according to standard protocols. The slides were scanned for digital imaging using a slide scanner (MoticEasyScan Pro 6, Motic Corp., Hong Kong).

### 4.4. Library Preparation and Sequencing

Tissues were collected at one day post-exposure. Total RNA was extracted from the hippocampus using TRIzol™ reagent (Thermo Fisher Scientific, Waltham, MA, USA). The quality of the total RNA recovered was assessed using an Agilent 4200 TapeStation system (Agilent Technologies, Santa Clara, CA, USA). After removing undesired ribosomal RNA (rRNA) using a RiboCop rRNA Depletion kit (Lexogen GmbH, Vienna, Austria), libraries were constructed using a NEBNext Ultra II Directional RNA Library Prep kit (New England BioLabs Inc., Ipswich, MA, USA) according to the manufacturer’s instructions. The enrichment of the libraries was carried out using PCR, and the libraries were assessed using an Agilent High Sensitivity DNA kit (Agilent Technologies, Santa Clara, CA, USA) on an Agilent 2100 bioanalyzer (Agilent Technologies, Santa Clara, CA, USA) to evaluate the mean fragment size. The constructed libraries were 101 bp paired-end sequenced using a NovaSeq 6000 system (Illumina, San Diego, CA, USA). Sequencing depth and read mapping statistics are provided in Table S2.

### 4.5. Differential Gene Expression Analysis and Functional Annotation

Quality control of raw sequencing data was performed using FastQC v0.11.9 [51]. Adapter contamination and low-quality reads (Q < 20) were removed using FASTX_Trimmer v0.0.14 [52] and BBMap v38.90, respectively [53]. The filtered reads were aligned to the reference genome using TopHat v2.1.1 [54]. The resulting FASTQ files have been deposited in the Korea BioData Station (K-BDS) [KAP241018] and will be made publicly available on 1 January 2028. Transcript abundance for genes, isoforms, and lncRNAs was estimated using fragments per kilobase of transcript per million mapped reads (FPKM) using Cufflinks v2.2.1 [54]. The resulting transcript expression data were imported into ExDEGA. ExDEGA provides comprehensive expression profiles, including (i) per-sample raw read counts, (ii) log_2_-transformed normalized expression values (“Normalized data (log_2_)”) calculated using counts per million values scaled by the trimmed mean of M-values normalization method implemented in edgeR, and (iii) group-wise average expression values calculated from these normalized values (Tables S3 and S4). Differential expression analysis was conducted manually within the GraphicPlus module of ExDEGA by applying researcher-defined criteria. Specifically, transcripts were considered significantly differentially expressed mRNAs and lncRNAs if they fulfilled all three criteria: (i) absolute log2 fold change ≥ 1 (equivalent to fold change ≥ 2.0), (ii) log_2_ normalized expression ≥ 2 in at least one experimental group, and (iii) raw *p*-value < 0.05, calculated using an unpaired two-tailed Student’s *t*-test implemented in base R v4.2.2. For all transcripts that satisfied these three filters, raw *p*-values were subsequently adjusted for multiple testing using the FDR correction implemented in base R. Heatmaps and volcano plots for visualization of differentially expressed transcripts were generated using ExDEGA (Ebiogen Inc., Seoul, Republic of Korea). GO enrichment analysis and KEGG pathway enrichment analysis were performed using DAVID Bioinformatics Resources (https://davidbioinformatics.nih.gov/tools.jsp, accessed on 11 January 2025). GO terms from all three categories—BP, cellular component (CC), and molecular function (MF)—were included. KEGG pathway analysis was used to identify signaling and metabolic pathways associated with the differentially expressed mRNAs.

### 4.6. qRT-PCR

qRT-PCR was performed to validate the expression of mRNAs and lncRNAs identified as differentially expressed in total RNA-seq analyses. Hippocampal samples were collected from each experimental group as follows: control (Con, n = 5), cannabidiol (CBD, n = 5), and delta-9-tetrahydrocannabinol (THC, n = 5). In addition, selected mRNA and lncRNA targets were further analyzed at 3 and 14 days post-exposure to assess time-dependent expression changes following a single vaping exposure. Total RNA was extracted using TRIzol™ reagent (Thermo Fisher Scientific, Waltham, MA, USA), and cDNA was synthesized using PrimeScript™ Reverse Transcriptase (TaKaRa, Shiga, Japan) according to the manufacturer’s protocol. Gene expression levels were normalized to that of Gapdh, and relative expression differences between the experimental and control groups were calculated using the 2^−ΔΔCT^ method. The primers used for amplification of candidate genes are listed in Table S5. Technical procedures for qRT-PCR have been described in detail previously [55].

### 4.7. mRNA-lncRNA Co-Expression Network

To explore potential regulatory relationships between differentially expressed mRNAs and lncRNAs, a trans-regulatory interaction analysis was conducted. Predicted binding interactions were identified using RIsearch with a free energy threshold <−30 kcal/mol to select highly confident base-pairing candidates. Putative binding interactions between lncRNAs and mRNAs were predicted using RIsearch, with a free energy threshold set to <−30 kcal/mol to ensure high-confidence base-pairing candidates. Co-expression relationships were evaluated by calculating Spearman correlation coefficients, and only mRNA–lncRNA pairs with Spearman ρ ≥ 0.95 were retained for further analysis. A co-expression interaction network was constructed and visualized using Cytoscape (v3.10.3; https://cytoscape.org). To further support the network findings, Pearson correlation analysis was performed using Python (version 3.13.2). In addition, a correlation heatmap was generated using custom Python scripts with the Seaborn v0.12.2 and Matplotlib v3.7.1 libraries. The heatmap illustrates the strength and direction of pairwise correlations between differentially expressed lncRNAs and mRNAs, with the color scale representing correlation coefficients ranging from −1 (perfect negative correlation) to 1 (perfect positive correlation).

### 4.8. Statistical Analysis

All statistical analyses were performed using GraphPad Prism (version 9.5.1; GraphPad Software, San Diego, CA, USA) and Python (version 3.13.2) with relevant statistical libraries. Quantitative data are presented as mean ± standard error of the mean (SEM). Differences between groups were evaluated using unpaired *t*-tests and Mann–Whitney *U* tests for normally distributed variables and non-parametric variables, respectively. For time-course analyses of gene expression at days 1, 3, and 14 post-exposure, two-way ANOVA was performed with treatment and time as factors, followed by Bonferroni’s multiple comparison test. A *p*-value < 0.05 was considered statistically significant. Correlation analyses for mRNA–lncRNA co-expression networks were conducted using both Spearman and Pearson correlation coefficients. Only gene pairs with a Spearman correlation coefficient (ρ) ≥ 0.95 were retained for network construction.

## Data Availability

The datasets generated and analyzed in this study are available from the corresponding author upon reasonable request.

## Supplementary Materials

The following supporting information can be downloaded at https://www.mdpi.com/article/10.3390/ijms26157106/s1.

## Abbreviations

The following abbreviations are used in this manuscript:

CBD	Cannabidiol
THC	Delta-9-tetrahydrocannabinol
lncRNAs	Long noncoding RNAs
EVALI	E-cigarette, or vaping, product use associated lung injury
qRT-PCR	Quantitative RT-PCR
H&E	Hematoxylin and eosin
ExDEGA	Excel-based differentially expressed gene analysis
GO	Gene ontology
KEGG	Kyto Encyclopedia of Genes and Genomes
BP	Biological process
PRC1	Polycomb repressive complex 1
PG	Propylene glycol
VG	Vegetable glycerin
rRNA	Ribosomal RNA
K-BDS	Korea BioData Station
FPKM	Fragments per kilobase of transcript per million mapped reads
CC	Cellular component
MF	Molecular function
