# Supplementary material for: Large-Scale Profiling of Coding and Long Noncoding Transcriptomes in the Hippocampus of Mice Acutely Exposed to Vaporized CBD or THC
Source: Int J Mol Sci. 2025 Jul 23;26(15):7106. doi: 10.3390/ijms26157106 (PMC12346642; doi:10.3390/ijms26157106)
Supplement: Supplementary file 1 [file ijms-26-07106-s001.zip › Table S2.pdf]

Table S2. Summary of sequencing depth and mapping statistics for each sample

Group	Sample		Total Reads	Total Bases	Clean Reads	Overall Mapping Rate (%)
Control	1	O1M_BH1	61,643,012	6,225,944,212	59,161,160	95.13
	2	O1M_BH2	62,084,926	6,270,577,526	59,711,964	94.96
	3	O1M_BH4	61,676,848	6,229,361,648	59,823,722	94.76
	4	O1M_BH5	64,012,550	6,465,267,550	62,530,172	95.00
	5	O1M_BH6	64,049,770	6,469,026,770	62,294,716	95.13
THC	6	A1TM_BH1	63,422,556	6,405,678,156	61,275,858	96.04
	7	A1TM_BH2	61,422,840	6,203,706,840	59,751,702	94.85
	8	A1TM_BH3	61,629,396	6,224,568,996	60,005,116	95.12
	9	A1TM_BH4	63,293,356	6,392,628,956	61,659,544	95.88
	10	A1TM_BH5	62,384,168	6,300,800,968	60,942,772	95.24
CBD	11	A1DM_BH1	62,115,280	6,273,643,280	60,721,576	95.24
	12	A1DM_BH2	63,391,272	6,402,518,472	61,962,826	95.75
	13	A1DM_BH3	63,344,460	6,397,790,460	62,040,440	95.24
	14	A1DM_BH4	62,282,578	6,290,540,378	60,857,276	93.96
	15	A1DM_BH5	62,918,784	6,354,797,184	61,194,772	94.50
