# Supplementary material for: Large-Scale Profiling of Coding and Long Noncoding Transcriptomes in the Hippocampus of Mice Acutely Exposed to Vaporized CBD or THC
Source: Int J Mol Sci. 2025 Jul 23;26(15):7106. doi: 10.3390/ijms26157106 (PMC12346642; doi:10.3390/ijms26157106)
Supplement: Supplementary file 1 [file ijms-26-07106-s001.zip › Table S5.pdf]

Table S5. Primers used in qRT-PCR

Gene	Forward (5'-3')	Reverse (5'-3')	Amplicon size (bp)
GAPDH	AGG TCG GTG TGA ACG GAT TTG	TGT AGA CCA TGT AGT TGA GGT CA	123
L3mbtl1	GGCACACTGACATGGAGATAC	GTAGGAATAATGAAGGCGGTTGT	130
Sv2b	AGGTATCGGGACAACTATGAGG	GCCTTCTGTAACATCGCTCTGT	102
Wnt7a	TGAACTTACACAATAACGAGGCG	GTGGTCCAGCACGTCTTAGT	112
Camk2b	GCACGTCATTGGCGAGGAT	ACGGGTCTCTTCGGACTGG	103
Grin2a	ACGTGACAGAACGCGAACTT	TCAGTGCGGTTCATCAATAACG	100
Gria3	ACCATCAGCATAGGTGGACTT	ACGTGGTAGTTCAAATGGAAGG	128
Camk2g	ACCGACGACTACCAGCTTTTC	GCAGCATATTCCTGCGTAGATG	95
Zhx2	ATGGCAAGCAAACGGAAATCT	TCCTTTGTCACATCGGACTGT	134
Dzip1	AGGTCAAGGGGATGTTTATGAGG	GGGGACACGGAAGATGGTC	165
Cdk16	AGATAAGACCAATGGTGTCCCT	CACTCTCCCCATCAGATCCCA	185
Rab3gap1	CCCGAGTCCGAAGTGTTTGAG	TTCCCAGAGAGGGTCCAATCA	118
Arhgap21	GCTCAGTGAGAATTTGGGAACA	GTCTTAAAGGCTGTGGCGTCT	173
Egr3	CCGGTGACCATGAGCAGTTT	TAATGGGCTACCGAGTCGCT	110
Erc2	ATGTACGGGAGCGCAAGAAC	GGCTGCATTAAGGGACTGGATA	165
Golga2	GACTACAGTCCTCCCGACAG	CGCTCTTTGGTTAGGTCCTTGT	109
Rapgef1	AAAGCAGACTCTCAGCGTTCT	TCTCAGGAATCTTCGACACCTC	130
Drd1	TTTTGGCCCTTTGGGTCCTT	GTCCACGCTGATCACACAGA	96
Drd2	ACCTGTCCTGGTACGATGATG	GCATGGCATAGTAGTTGTAGTGG	105
Gnal	GCCAACAAAAAGATCGAGAAGC	GTTGAAGCCATTGACGTGCAG	144
Adcy5	AACGCCAAGCAGGAGGATATG	CCCCGAGGATCTTAATCCGTAA	208
Rgs9	TTCCCCGAGTCGCTTCATC	CTGGGGTCTTGAGTGGTCT	178
Pde7b	AAGGCACATGCTCTCCAAAGT	GTTACCAGACTGTTCCCATTTGT	84
Penk	GAGAGCACCAACAATGACGAA	TCTTCTGGTAGTCCATCCACC	166
Pde10a	AGGATACGAATATGCAGGGAGT	CCGTCGGCTTTTGTGGCTAT	133
Tac1	TTTCTCGTTTCCACTCAACTGTT	GTCTTCGGGCGATTCTCTGC	145
Adora2a	GCCATCCCATTCGCCATCA	GCAATAGCCAAGAGGCTGAAGA	122
Six3	GCTCCCGGCTTCTCTTACC	CGGCGAAGTTTGGCAACAAG	97
NONMMUT069014.2	GTGCTGTGGGTTTCTGGGAT	ATTCTGGGAATGGGCAGGTC	165
NONMMUT033147.2	CAGTCAACTCTGGTCGCCAT	TGAGCCACAGCCATCTTCAG	179
NONMMUT072606.2	GAGACAGAGAGCACGCAACT	GGGAGAGCAGCTGTTGACAT	134
NONMMUT034199.2	AGAGATGACAAGGCAGCACC	CAATTTGCACCCAGGAAGCC	110

NONMMUT072606.2	GAGACAGAGAGCACGCAACT	GGGAGAGCAGCTGTTGACAT	134
NONMMUT008020.2	TGCATCCAGATCAAGACCTCAC	TGTCAGGTGATCTACCCTTTGG	155
NONMMUT085523.1	CCGTCTCTCCTCCATCCAGT	CCTGGGTGTGGTGGTACAAA	128
NONMMUT123548.1	TTCAGCCTCAAGGCATCTCG	TGAGTCTTCCTGAGAGCCCA	183
NONMMUT061628.2	TGCTGATGCCTCTTTCCTGT	GTCAAGTGGCAGCAAGGGAA	138
NONMMUT019734.2	CCACAGCATTTGCCAGTTGT	ACTCTCTGGGGATGGGGATAG	152
NONMMUT028153.2	ATCCCGGGCAGCAAAATACA	CTGGTCACTCTTCAGGCTGG	189
NONMMUT057101.2	GACACATGGGAGCACTGTCA	TAGCCTGCTGTTTCCCCTTG	174
NONMMUT004928.2	AAGCAATCACGAGCATCCGA	TCTTGGGGAGAGAGGAGAGC	113
NONMMUT030952.2	ACTTGGCGATCGAGGTTTGG	CCTCCAAAGCGGCTAGGAAA	144
NONMMUT057185.2	CTCAGCTCTCACTTGCCACA	TGGTCCCCACCATCATCTCT	186
NONMMUT093973.1	CAACCAGGTGCCAGAAGTGA	CTACAGCTTGGGAGTTGCCT	99
NONMMUT021952.2	CAAAGGTCTTGGGCTGTGGA	CTCTGGGAGCTGGAAGAAGC	125
NONMMUT033944.2	TCCGGACCTGAAATGTGTCTT	AGACTGACAGAAGATGGGCA	200
NONMMUT071342.2	TCAGAGTTCAGAGCCCAGGA	AGTTGGGGACAGCAAGAGTG	136
